# Supplementary material for: Access to Vaccines in Floodplains and Hard-to-Reach Areas of the Brazilian Amazon: The Contribution of Street-Level Bureaucrats and the Use of Social Technologies
Source: Int J Environ Res Public Health. 2025 Apr 25;22(5):680. doi: 10.3390/ijerph22050680 (PMC12111495; doi:10.3390/ijerph22050680)
Supplement: Supplementary file 1 [file ijerph-22-00680-s001.zip › ijerph-3462674-supplementary.pdf]

Supplementary Material S1.

Professionals of the National Immunization Program and their respective functions

PROFESSIONAL	ASSIGNMENTS
Immunization Coordinator	• Technical responsible for mapping the Municipality according to the population and organizing their respective vaccination rooms;<br>• Request immunological and supplies from the State NIP according to the demand of the Municipality both in Campaigns and in routine Vaccination;<br>• Establish partnerships with public agencies, especially schools, to ensure the dissemination of the importance of the population always being immunized, including requesting a list of students to schedule vaccination in the classrooms (in these cases it is necessary to obtain authorization from those responsible and presentation of the vaccine card);<br>• Conduct training for Vaccinators and Community Health Agents so that they are able to check the vaccine card in their home visits, thus carrying out and helping in the active search for those who are absent so that they can be immunized as soon as possible by vaccinators in the Family Health Strategy team;<br>• Supervise the Information Systems of the Vaccinated of the Municipality to follow and monitor the indicators for the achievement of the Vaccination goals recommended by the Ministry of Health;<br>• Carry out technical visits to the vaccination rooms to survey both structural and human resources needs in order to, when necessary, obtain the support of local management;
Nursing technicians (vaccinators)	• Work in the Vaccine Rooms and carry out home immunization of users with an "incomplete vaccination schedule", especially children, in order to guarantee the minimum indicators that are obtained by the administration of the Pentavalent, MMR, Pneumococcal and VIP vaccines (vaccine against Poliomyelitis);<br>• Keep the Vaccine Rooms in order and monitor the temperature of the Immunobiologicals to ensure their effectiveness;<br>• Advise users about the importance of each vaccine, its possible adverse events and what to do in case of these reactions;
Community Health Agent	• Carry out home visits to disseminate and guide on the importance of everyone being immunized both in Campaign and Routine Vaccines; and read the Vaccine cards to detect possible absences and ensure the completion of the vaccination schedule. They are responsible for referring these users to the vaccination room in their area, or requesting that the vaccination team attend the site to administer the missing vaccines;
Doctors, nurses, dentists, and all professionals of the Family Health strategy	• These professionals are fundamental and strong allies in the immunization process because they are the ones who guide the population in the environment of the Health Units, clarifying doubts and referring them to complementary services. They help to screen users, thus contributing to a more humanized and assertive service.

STAKEHOLDER	PERFORMANCE AND ATTRIBUTIONS
Boatmen and boat assistants	They are professionals with experience in driving small and medium-sized boats, hired by health managers to transport immunizers and immunization teams to floodplain areas and areas of difficult access. The assistants are professionals with less

	time working in the area of river transport who work helping veterans.
Community leaders	Influential people in the communities and villages who belong to different professional categories such as fishermen, merchants, farmers, among others (among whom are shamans and other indigenous leaders). They are a link between government agents and health service users. They work to raise awareness, motivate and articulate users, being collaborators and supporters of government agents to ensure the population's adherence to vaccines, creating logistical means for people to attend the vaccination rooms.
Meteorological Service Technicians	Professionals in the field of meteorology who work with health managers in technical information. They maintain direct daily contact with immunization teams, providing real-time information through radios or mobile phones. They are important to ensure that navigation to floodplain areas is safe and that it occurs on time. These professionals are also part of the support network of the immunization teams, often being called by them in situations of accidents or complications on the way of the teams.
Road drivers	They are responsible for transporting the immunizers on all road stretches. In locations served by road or road and river, these professionals have a great responsibility, as they transport, in addition to the immunizers, all health professionals and teams of cooks, assistants and their work equipment. In general, these professionals are part of the staff of the municipal health department.
Cooks	They are responsible for producing meals for the teams involved. They take care of the safety and handling of the inputs used in the production and service of meals, whether on board the vessels or in the locations where immunization takes place. In general, these professionals are part of the staff of the municipal health department.

SOURCE: Organized by the authors based on documents from the National Immunization Program of the Ministry of Health and based on the daily practice of the immunization teams
